# Killed whole-HIV vaccine; employing a well established strategy for antiviral vaccines

**DOI:** 10.1186/s12981-017-0176-5

**Published:** 2017-09-12

**Authors:** C. Yong Kang, Yong Gao

**Affiliations:** Department of Microbiology and Immunology, Schulich School of Medicine and Dentistry, The University of Western Ontario, London, ON N6G 2V4 Canada

## Abstract

The development of an efficient prophylactic HIV vaccine has been one of the major challenges in infectious disease research during the last three decades. Here, we present a mini review on strategies employed for the development of HIV vaccines with an emphasis on a well-established vaccine technology, the killed whole-virus vaccine approach. Recently, we reported an evaluation of the safety and the immunogenicity of a genetically modified and killed whole-HIV-1 vaccine designated as SAV001 [1]. HIV-1 Clade B NL4-3 was genetically modified by deleting the *nef* and *vpu* genes and substituting the coding sequence of the Env signal peptide with that of honeybee melittin to produce an avirulent and replication efficient HIV-1. This genetically modified virus (*gm*HIV-1_**NL4-3**_) was propagated in a human T cell line followed by virus purification and inactivation by aldrithiol-2 and γ-irradiation. We found that SAV001 was well tolerated with no serious adverse events. HIV-1_**NL4-3**_-specific polymerase chain reaction showed no evidence of vaccine virus replication in participants receiving SAV001 and in human T cells infected in vitro. Furthermore, SAV001 with an adjuvant significantly increased the antibody response to HIV-1 structural proteins. Moreover, antibodies in the plasma from these vaccinations neutralized tier I and tier II of HIV-1 B, A, and D subtypes. These results indicated that the killed whole-HIV vaccine is safe and may trigger appropriate immune responses to prevent HIV infection. Utilization of this killed whole-HIV vaccine strategy may pave the way to develop an effective HIV vaccine.

## Background

Despite proactive HIV/AIDS education and availability of effective combination antiretroviral therapy (cART), the AIDS pandemic continues. WHO and UNAIDS estimate that close to 37 million people are currently living with HIV infection and approximately 40 million HIV related deaths have occurred since the discovery of HIV-1 [2]. However, vaccine development against HIV continues to struggle due to unresolved questions on the exact components of the human immune response that confer protection against HIV infection. Similar to multiple effective anti-viral vaccines, a safe, effective, and globally accessible HIV vaccine must be developed to end the AIDS pandemic.

## Past human clinical trials of HIV vaccines

The first human clinical trial of Vaxgen’s AIDSVAX was based on a recombinant HIV-1 gp120. This trial showed that 5.7% of 3330 vaccinated high-risk volunteers contracted HIV while 5.8% of the 1679 placebo control group contracted HIV [3]. The monomeric form of recombinant gp120 induced antibodies but failed to neutralize the virus. We now know that the native conformational epitopes of the trimeric form of gp120 are key to generating neutralizing antibodies [4, 5].

The second HIV vaccine human clinical trial was conducted by Merck. This trial was known as the STEP study or Phambili Trial which utilized a recombinant adenovirus 5 (Ad 5) vector carrying HIV-1 *gag*, *pol,* and *nef* genes [6]. Merck’s recombinant Ad 5 vaccine was neither effective in preventing infection nor in reducing viral loads. Trial results showed that 3.2% of 741 vaccinated volunteers became infected, but only 2.7% of the 762 individuals in the placebo group became infected with HIV-1 during the STEP study [6]. Merck discontinued STEP study in September 2007. The reason for the higher rate of infection among the vaccine group was believed to be a result of preexisting Ad 5 antibodies in these subjects.

In September 2009, the US National Institutes of Health announced the results of a Phase III human clinical trial, known as RV144, conducted in Thailand. RV144 consisted of a recombinant Canary poxvirus carrying HIV-1 *gag, pol* and *env* genes as a prime vaccine known as ALVAC developed by Sanofi Pasteur followed by a boost vaccine of gp120 based AIDSVAX. The results of this third human trial showed that 51 of 8197 (0.6%) vaccinated individuals became infected while 74 of 8198 (0.9%) placebo control group contracted the HIV [7]. Thus, the vaccine cut the risk of infection by 31.2% after 42 months [8–10]. While the RV144 vaccine did not induce any broad spectrum neutralizing antibodies, it did induce non-neutralizing antibodies presenting potent antibody-dependent cellular cytotoxicity (ADCC) activity which could have decreased transmission [11, 12]. Even though these results showed potential for an HIV vaccine, it was not enough to overcome the HIV epidemic and develop a commercialized product. There will be a further large scale HIV vaccine trial conducted in South Africa with this vaccine. Indeed, the HVTN 702 trial will be an extension of the RV144 clinical trial using the canarypox-based vaccine with bivalent gp120 protein. This study will enroll 5400 HIV-uninfected men and women volunteers (NIAID, NIH). It will be interesting to see whether or not the HVTN702 trial will meet expectations.

Finally, the fourth vaccine trial used a combination of a DNA-based vaccine followed by a recombinant Ad 5-based vaccine boost (known as the HVTN 505 trial conducted by the US NIAID) which showed that 41 of 1250 individuals (3.3%) in the vaccine group and 30 of 1244 individuals (2.4%) in the placebo group contracted HIV during the trial [13]. Like Merck’s STEP trial, this DNA priming followed by a recombinant Ad 5 boosting combination vaccine also had a higher rate of infection among the vaccinated group compared to the non-vaccinated control group.

## Three types of commercially available anti-viral vaccines

There are three types of commercially available vaccines. These include attenuated live virus vaccines, recombinant protein based subunit vaccines, and killed whole-virus vaccines. Although there have been many successful live attenuated viral vaccines developed against many human and animal viral diseases, the attenuated live virus vaccine strategy is not an option for HIV, because the proviral DNA of live attenuated HIV will be integrated into the host chromosomal DNA to establish a persistent infection.

With respect to the subunit vaccine strategy, two successful viral subunit vaccines have been developed. The 22 nm virus-like particles (VLP) of human hepatitis B virus S antigen and the L1 protein-based VLP of the human papilomavirus vaccine are effective subunit vaccines with proven efficacy. VaxGen has already attempted recombinant gp120-based vaccine for HIV. However, the AIDSVAX of VaxGen failed to provide protective immunity due to its monomeric form of gp120 which cannot generate neutralizing antibodies. Thus, scientists have tried to express the trimeric form of gp120 in order to induce broadly neutralizing antibodies [14, 15].

The third commercially available antiviral vaccine is the killed-whole virus vaccine. This vaccine strategy has prevented many viral diseases such as polio, influenza, rabies, and hepatitis A [16–19]. In addition, there are at least 16 licensed killed whole-virus vaccines available to protect against various viral diseases in animals. However, the killed whole-virus vaccine approach has been neglected for HIV vaccines due to risks associated with incomplete inactivation of HIV and other technical challenges. Interestingly, only one group (Remune) previously attempted a killed whole-HIV vaccine strategy. However, the lack of gp120 on the virion surface of the Remune vaccine and other safety concerns led to the discontinuation of Remune’s project [20].

## Design of killed whole-HIV vaccine

The killed whole-HIV-1 vaccine approach has great merit as it has the potential to present multiple viral antigens to the immune system in their native conformation. Thus, two groups have suggested that it is time for another look at inactivated (killed) HIV vaccine for prevention of HIV infection [21, 22]. The challenges in developing a killed whole-HIV vaccine include the lack of techniques for high level virus production and the safety issues associated with virus production in large quantities. We overcame these problems by deleting the *nef* gene which contributes to AIDS pathogenesis [23–26] and substituting the envelope glycoprotein signal peptide gene to achieve high level virus replication [27, 28].

We have shown that the natural HIV-1 *Env* protein signal peptide is highly inefficient for intracellular processing of HIV glycoproteins [27], however replacement of the HIV-1 *Env* protein signal peptide with the signal peptide of honeybee melittin results in enhanced protein expression, glycosylation, folding, intracellular transport, and processing [27, 28]. Furthermore, due to the overlapping reading frame of the *vpu* gene with that of the HIV-1 natural signal peptide gene, our killed whole-HIV-1 (SAV001) has a deletion of the *vpu* gene as well. The genetically modified HIV-1_**NL4-3**_ (*gm*HIV-1_**NL4-3**_) was propagated in the A3.01 human T cell line, purified through sucrose gradient and inactivated by aldrithiol-2 [29, 30] and 30 kGy of gamma irradiation [31]. This purified SAV001 showed intact virion morphology under electron microscopy and its capability to aggregate AA2 and A3.01 human T lymphocytes in culture indicate that functional gp120 peplomers are present on the purified virions [1].

## Inactivation of HIV-1

The major concern for the killed whole-HIV vaccine is the incomplete inactivation of HIV. One viable HIV virion during the virus inactivation process is indeed one too many for a killed whole-virus vaccine. To ensure the safety of the vaccine, we deleted the *nef* gene for attenuation, and used both chemical (aldrithiol-2) and physical (γ-irradiation) inactivation to provide complete killing. The test for in vitro replication of SAV001 using sensitive methods showed that virus replication was absent even after ten consecutive passages in human T lymphocytes [1].

Furthermore, following immunization, highly sensitive, external-nested polymerase chain reaction (PCR) amplifications using vaccine virus-specific primers on the viral RNA in plasma confirmed the lack of any virus genome originating from the vaccine. In contrast, the external-nested PCR amplification by generic HIV-1 primer sets amplified HIV-1 RNA despite undetectable viral loads by the less sensitive Roche Amplicor assay (<50 copies/ml). Pyrosequencing of these PCR products before and after immunization with SAV001 again confirmed the lack of HIV-1 NL4-3 in over 71,000 HIV sequence reads and the presence of only patient-specific HIV-1 strains. These results provide very strong evidence that aldrithiol-2 treatment followed by gamma irradiation (30 kGy) completely inactivate HIV-1 and ensure the safety of the vaccine recipients [1].

## Immunogenicity of the killed whole-HIV-1 vaccine

Despite the complete inactivation of *gm*HIV-1_**NL4-3**_ virus used in our study, we only enrolled HIV-1 positive asymptomatic volunteers based on discussions with the US FDA. Therefore, even though the study was designed to evaluate safety and tolerability, the nature of the study limited our ability to assess the immunogenicity of the vaccine formulations. Previous studies have shown that HIV-1 infection elicits antibody responses to proteins encoded by HIV-1 *gag, pol* and *env* genes, and the antibody response to various proteins appears at different stages of infections [32]. As expected, most of the subjects in our study had a high baseline antibody titer against viral structural proteins. However, with a single SAV001 intramuscular vaccination, the humoral immune response was significantly increased by boosting the secondary anti-HIV antibody responses in vaccinated groups suggesting a strong immunogenicity of the SAV001 vaccine.

The importance of the design of immunogens, especially the trimeric form of the envelope glycoproteins, capable of inducing broadly neutralizing antibodies is the major focus of current HIV-1 vaccine research [33, 34]. Even though our killed whole-*gm*HIV-1_**NL4-3**_ virus was purified and completely inactivated through combined chemical and physical inactivation processes, its Env glycoproteins were not readily shed and were still functional. Our results suggested that SAV001 vaccine can mimic natural infection through its native viral structure, especially the native form of envelope glycoprotein which is crucial for eliciting broadly neutralizing antibodies. Indeed, when compared to placebo control, our data supported this hypothesis as SAV001 was able to stimulate anti-gp120 antibodies in plasma that could recognize trimeric Env glycoproteins at the surface of infected cell and mediate ADCC. Although most of subjects in our trial were infected with HIV-1 subtype B, sera from these subjects neutralized not only subtype B but also subtypes D and A which is consistent with the notion that HIV-1 superinfection is not a common event [35–37]. Thus, an HIV-1 vaccine based on one subtype may be able protect against infections of other subtypes.

## Conclusion

Vaccination with SAV001, the genetically modified and killed whole-HIV-1 vaccine, could enhance humoral immune responses including broadly neutralizing antibody production in HIV-negative individuals. Therefore, SAV001 represents a promising starting point for the development of a safe and effective prophylactic HIV-1 vaccine using the killed whole virus approach. This approach could be easily adaptable to include different subtypes of HIV-1.
